# Effectiveness of hydrogen peroxide as auxiliary treatment for hospitalized COVID-19 patients in Brazil: preliminary results of a randomized double-blind clinical trial

**DOI:** 10.4178/epih.e2021032

**Published:** 2021-05-01

**Authors:** Marielle Bazzo Di Domênico, Henrique Cesca, Thales Henrique Jincziwski Ponciano, Renan Brandenburg dos Santos, Ulysses Lenz, Vinícius Picoli Antunes, Vinicius Webber Godinho, Kauê Collares, Pedro Henrique Corazza

**Affiliations:** Graduate Program in Dentistry, Dental School, University of Passo Fundo, Passo Fundo, Brazil

**Keywords:** Hydrogen peroxide, COVID-19, SARS-CoV-2, Mouthwashes, Nasal sprays

## Abstract

**OBJECTIVES:**

To evaluate the effectiveness of hydrogen peroxide (H_2_O_2_) in the form of mouthwash and nasal spray as an auxiliary treatment for coronavirus disease 2019 (COVID-19).

**METHODS:**

Forty hospitalized patients who tested positive for severe acute respiratory syndrome coronavirus 2 using a reverse-transcription polymerase chain reaction test were evaluated. They were randomly divided into an experimental group (n= 20; gargling with 1.0% H_2_O_2_ and nasal wash with 0.5% H_2_O_2_) or a control group (n= 20). The solutions were used for 7 days and the patients were monitored every 2 days, for a total of 8 days. At check-ups, patients were asked about their symptoms and possible adverse effects of the solutions. The presence and severity (mild, moderate, or severe) of symptoms were recorded. Data were compared using the Student test and the Fisher exact test (α= 0.05).

**RESULTS:**

There was no significant difference between the 2 groups in the length of hospital stay (p= 0.65). The most frequent symptom on day 0 was coughing (72.0% in the experimental group and 76.5% in the control group), which abated over time. There was no significant difference between the groups in the evaluated symptoms. Most (75.0%) of the patients in the experimental group presented a reduction in dyspnea between days 0 and 2. Few patients reported adverse effects from the use of the solutions.

**CONCLUSIONS:**

H_2_O_2_ as a mouthwash and nasal spray is safe to use. There is insufficient evidence to demonstrate that H_2_O_2_ is effective as an auxiliary treatment for hospitalized COVID-19 patients.

## INTRODUCTION

Since its emergence in Wuhan, China in December 2019, severe acute respiratory syndrome coronavirus 2 (SARS-CoV-2) has become one of the biggest problems of the modern era. The reasons for its exponential growth include its high transmissibility between individuals, whether directly (coughing, sneezing, and inhaling saliva droplets), or indirectly (contact with contaminated surfaces) [1]. Moreover, the very high number of undocumented and asymptomatic cases (potentially reaching 79% of cases) also increases its transmission [2]. The median incubation period of the virus is estimated to be approximately 5 days (between 2 and 7 days), and 97.5% of the patients who develop symptoms will do so within 11.5 days of infection [3,4]. The median interval from symptom onset to hospitalization is 7 days (interquartile range, 3-9) [5]. According to a systematic review [6] that included 24,410 adults with coronavirus disease 2019 (COVID-19), the most prevalent symptom was fever (78%), followed by coughing (57%) and fatigue (31%). Other less frequent symptoms were observed, such as hyposmia (25%), dyspnea (23%), myalgia (17%), chills (17%), wheezing (17%), headache (13%), sore throat (12%), arthralgia (11%), vertigo/dizziness (11%), mental confusion (11%), and diarrhea (10%). Among hospitalized patients, the most common symptoms were fever (up to 90% of patients), dry cough (60-86%), shortness of breath (53-80%), fatigue (38%), nausea/vomiting or diarrhea (15-39%) and myalgia (15-44%) [2,3,7-9]. The complications of COVID-19 include cardiac, brain, lung, liver, kidney, and coagulation system dysfunction. COVID-19 can also trigger cardiomyopathy, ventricular arrhythmias, and hemodynamic instability [10]. The most common comorbidities of hospitalized patients are hypertension (present in 48-57% of patients), diabetes (17-34%), cardiovascular disease (21-28%), chronic lung disease (4-10%), chronic kidney disease (3-13%), malignancy (6-8%) and chronic liver disease (< 5%) [5,8,11].

Starting on the first days of contact, the virus remains in patients’ upper airway, being detected in the saliva of 91.7% of those infected [12]. The oropharynx and nasopharynx have high viral loads [13,14], and are the main site of replication and elimination of the virus during the course of the disease [15]. These regions are directly associated with the evolutionary process of COVID-19. Zou et al. [14] analyzed the viral load in the nose and throat of samples obtained from symptomatic and asymptomatic patients. Higher viral loads were detected soon after the onset of symptoms, and the loads were higher in the nose than in the throat. According to Herrera et al. [16], the oral viral load of SARS-CoV-2 was associated with the severity of COVID-19 and, therefore, a reduction of the oral viral load may be associated with a decrease in disease severity. Similarly, a decrease in the oral viral load would decrease the amount of virus expelled and reduce the risk of transmission. Thus, antiseptic mouthwashes could potentially be beneficial for infected patients, but clinical studies are needed to confirm this possibility.

Hydrogen peroxide (H_2_O_2_) in low concentrations has been used for several purposes. It has been proven to be effective in decontaminating surfaces [17,18], and has recently been used to decontaminate N95 respirators for reuse [19]. In dentistry and otorhinolaryngology, H_2_O_2_ has been used for tooth whitening and as an antiseptic and cleaning agent [20,21]; mucosal irritation is rare or absent, even after long periods of use [22,23]. The advantages of H_2_O_2_ include easy access, low cost, and a long history of use in dentistry. However, direct and prolonged contact (more than 30 minutes) with the mucosa can result in irritation [22,23], and high doses must be avoided, especially in patients with cardiovascular disorders [24]. H_2_O_2_ disrupts the lipid membranes of some viruses through the action of oxygen free radicals. Studies report that coronavirus 229E and other enveloped viruses can be inactivated at concentrations of around 0.5% [17,18]. Caruso et al. [23] suggested using a mouthwash with H_2_O_2_ soon after the beginning of the first symptoms, or a positive diagnosis for SARS-CoV-2, for disinfection of the oral cavity, and nasal washing with spray twice a day.

Thus, the objective of the present study was to evaluate the effectiveness of H_2_O_2_ in the form of a mouthwash (1.0%) and nasal spray (0.5%) as an auxiliary treatment for hospitalized COVID-19 patients. The hypothesis was that the treatment would be effective for reducing the symptoms related to the disease.

## MATERIALS AND METHODS

### Study design

This study was a randomized, double-blind, parallel, placebocontrolled clinical trial to assess the effectiveness of gargling and nasal wash with H_2_O_2_ to reduce COVID-19 symptoms in adult hospitalized patients.

**Figure 1.**
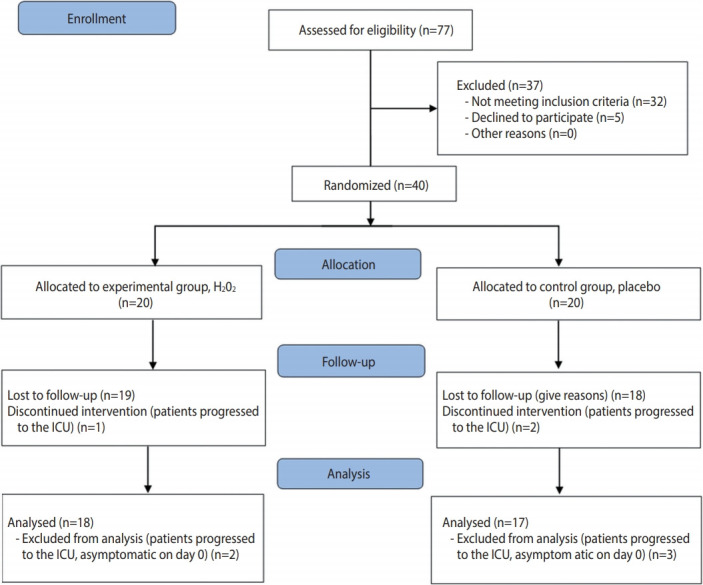
CONSORT (Consolidated Standards of Reporting Trials) flow diagram. H_2_O_2_, hydrogen peroxide; ICU, intensive care unit.

The study was registered in the Brazilian Registry of Clinical Trials (No. RBR-6sx3sz) and followed the CONSORT (Consolidated Standards of Reporting Trials) criteria for clinical studies (http://www.consort-statement.org/). The CONSORT flow diagram is presented in Figure 1.

### Patients

The eligible patients (n= 40) were admitted to the hospital with COVID-19 symptoms, and who were at least 18 years old and reverse-transcription polymerase chain reaction (RT-PCR) positive for SARS-CoV-2. During the research period (July and August 2020), there were 5,208 confirmed cases of COVID-19 in Passo Fundo.

The eligibility criteria were: having tested positive for SARS-CoV-2 and having received the diagnosis less than 3 days before the intervention, being hospitalized outside the intensive care unit, having the physical capacity to gargle and apply the nasal spray on their own, having moderate or mild COVID-19 symptoms, and agreeing to participate in the study.

### Randomization

Patients who met the eligibility criteria were randomly divided into experimental (gargling with 1.0% H_2_O_2_ and nasal wash with 0.5% H_2_O_2_, both associated with a mint essence) or control (placebo with deionized water associated with a mint essence, administered in the same way as the experimental group). Twenty patients were included in each group, without any predictability of allocation. Each group had specific letters for their representation. A randomization list was generated through a randomization website (https://www.random.org/). The randomization list was transferred to individual, sealed, opaque, and non-translucent envelopes. The envelopes containing a letter were given to the research team, who did not know the meaning of each letter. The patient drew his or her group’s envelope.

### Blinding

The person responsible for manipulating the solution and a study researcher, who was not involved in the solution’s distribution and assessment of outcomes, were aware of which letters corresponded to each group. Packages were prepared containing the appropriate treatments (H_2_O_2_ or placebo), all with the same appearance, differentiated only by the letter corresponding to the group. The participants, researchers responsible for the solution’s distribution and data tabulation, and the statistician were blinded.

### Researcher team training

The research team was trained before the intervention. An approach guide was created and provided to the 2 researchers who contacted the patients. Therefore, the initial questions, instructions for using the solutions, randomization, and approach on the other days were standardized. A communication channel via WhatsApp was created for contact between the researchers (HC and THJP) and the research coordinator (PHC).

### Interventions

The 2 groups (n= 20) of the study were: (1) Experimental (1.0% H_2_O_2_ for gargling and 0.5% H_2_O_2_ for the nasal wash): Patients gargled with a solution composed of 1.0% H_2_O_2_ and mint essence for 30 seconds, 3 times a day, for 7 days. One dose of the nasal spray was applied in each nostril, twice a day, for 7 days. The nasal solution was composed of 0.5% H_2_O_2_ and mint essence. (2) Control (placebo): The control group gargled and applied the nasal spray in the same way as described for the experimental group. The placebo solution was composed of distilled water and mint essence.

A 10-fold volumetric dilution of 3% H_2_O_2_ (pH= 3.40) was prepared for the experimental group. One liter of H_2_O_2_ was diluted in 2 L of deionized water and 20 mL of essence (liquid mint extract), obtaining a pH of 4.33 and a solution with 1.0% H_2_O_2_ for gargling. For the experimental nasal spray solution, 100 mL of the gargling solution was diluted in 100 mL of deionized water (pH= 4.55). For the placebo gargling, 20 mL of essence was added to every 3 L of deionized water. For the placebo nasal spray, 100 mL of the placebo for gargling was diluted in 100 mL of deionized water.

### Data collection

On day 0 (first contact), the patient was invited to participate in the research, and the kit composed of the gargling solution and nasal spray was provided. On the same day, individual variables were obtained from a questionnaire developed by the researchers. Socioeconomic and socio-demographic characteristics, comorbidities, and the patient’s symptoms at baseline were recorded. (Table 1).

### Outcomes

Patients were monitored in the hospital every 2 days, for 8 days, by the 2 trained researchers (for a total of 4 visits). If patients were discharged before the end of the survey, follow-up was carried out by phone. During follow-up visits, patients were asked about their symptoms with the question: “Do you have any of these symptoms? Fever, coughing, hyposmia, loss of taste, dyspnea, sore throat, or body pain?” If so, the severity of the symptom was asked (1, mild; 2, moderate; or 3, severe). The possible adverse effects of the solution were also recorded with the question: “Did you have any of these symptoms after using the solution? A burning sensation in your mouth, a burning sensation in your throat, food tasting unpleasant after use, the feeling of having a thick tongue, or a burning sensation in your nose?” If so, the severity of the symptom was asked (1, mild; 2, moderate; or 3, severe). For the clinical improvement variable, patients were considered to improve if, after 2 days of data collection, the patient did not present any of the COVID-19 symptoms evaluated in the study. The patient’s self-report was recorded in the form. Other clinical data were also recorded in the same form (discharge or transfer to the intensive care unit). All data were entered into an electronic database.

### Statistical analysis

Stata version 14 (StataCorp., College Station, TX, USA) was used for data analysis. A descriptive analysis was first performed to determine the relative and absolute frequency of patient characteristics. The time between the start of treatment with the solutions and discharge, in each group, was compared using the Student t-test (α= 0.05). The frequency of the symptoms—fever, coughing, hyposmia, loss of taste, dyspnea, sore throat, and body pain—was calculated on each day (0, 2, 4, 6, and 8). The proportion of individuals with relief from the symptoms of fever, coughing, dyspnea, and sore throat during days 0-2 and 2-4 were compared using the Fisher exact test (α= 0.05). The possible adverse effects of the solutions were compared graphically.

Efficacy analysis was performed on an intention-to-treat basis, including all the patients who had undergone randomization. The time to clinical improvement was assessed after all patients had reached day 8 and was portrayed by a Kaplan–Meier plot. Hazard ratios (HRs) with 95% confidence intervals (CIs) were calculated by means of a Cox proportional hazards model.

### Ethics statement

Ethical approval was obtained from the National Research Ethics Commission (CONEP, #4.071.153) and from clinics hospital, the hospital involved in the research. All patients (or their legal representative) approved and signed the informed consent form.

## RESULTS

When considering patients who discontinued the intervention and those who were asymptomatic on day 0 (2 in the experimental group and 3 in the control group), 35 patients were analyzed. Twenty-two (62.9%) were women and 13 (37.1%) were men. The predominant age of the patients included in the study was between 36 years and 59 years old (57.1%). The most prevalent comorbidities were hypertension (48.6%) and diabetes (28.6%). Table 1 shows the characteristics of the patients at baseline.

The average time between the beginning of treatment and discharge was 3.86± 1.60 days in the experimental group, and 4.15± 1.77 days in the control group; this difference was not statistically significant (p= 0.65).

The number of patients with each symptom on the evaluated days can be seen in Table 2. There were no reports of fever on day 0 among the patients in the present study. The most frequent symptom on day 0 was coughing (72.2% in the experimental group and 76.5% in the control group), which considerably abated over time. On day 0, 27.8% of the experimental group and 17.7% of the control group had a sore throat, which practically disappeared after day 2. The frequency of symptoms is shown in Table 2.

Fever was excluded from the symptom relief assessment. The proportions of individuals with relief from coughing, dyspnea, and sore throat are shown in Table 3. There was no significant difference between the groups using the Fisher exact test for the symptoms of coughing (p = 0.67), dyspnea (p = 0.15), and sore throat (p= 1.00). Seventy-five percent of the experimental group patients had relief for dyspnea between days 0 and 2 of the treatment.

**Figure 2.**
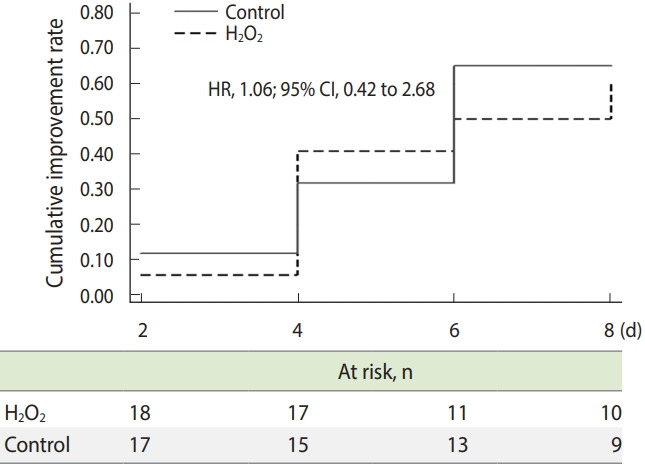
Time to clinical improvement in an intention-to-treat analysis. H_2_O_2_, hydrogen peroxide; HR, hazard ratio; CI, confidence interval.

Patients assigned to the H_2_O_2_ group did not present a different time to clinical improvement from that of patients assigned to the control group in the intention-to-treat population (median, 4 days vs. 4 days; HR for clinical improvement, 1.06; 95% CI, 0.42 to 2.68; p= 0.90) (Figure 2).

Table 4 shows the frequency of adverse effects reported by patients on days 2, 4, and 6 of the study. Few patients reported adverse effects associated with the use of the solutions during treatment. The most common effects were a burning throat on day 2 (22.2%), nasal burning on day 2 (16.7%), and the feeling of a thick tongue on day 4 (18.7%).

## DISCUSSION

The present study evaluated 35 symptomatic patients hospitalized with COVID-19, aiming to verify the effectiveness of a H_2_O_2_- based mouthwash and nasal spray for COVID-19 symptom relief and time of hospitalization. The homogeneity between both groups in most characteristics reflects the effectiveness of the randomization. Originally, 20 patients were included in each group, but 18 patients were ultimately analyzed in the experimental group and 17 patients were analyzed in the control group, due to exclusions after the allocation. The slightly uneven distribution can be considered a limitation of this study. Despite having a small sample, the present study was able to indicate many points to build upon that could serve as the basis for new experiments related to this topic. The difficulty of conducting controlled clinical trials with COVID-19 patients during the greatest epidemic of the modern era must be highlighted. Inpatients in the COVID-19 ward already undergo a strict process of infection control and must fill out many forms, which hinders their willingness to agree to participate in research. In addition, hospital officials evaluated the project in detail before the researchers had access to the patients. Furthermore, the results of RT-PCR tests for SARS-CoV-2 carried out through the Brazilian public health system take quite a long time to be released. Since it was established that only patients who recently tested positive would be included in the research, the average between the onset of symptoms and the start of using the solutions was 10.72± 2.68 days. This was a problem since some symptoms, such as fever, were no longer present in the sample. In further research building upon the present study, symptomatic patients at the hospital and receiving home treatment will be included. The issue regarding the time between symptoms and the start of treatment is being solved by giving the solutions to patients who sought care even before the test result was released. However, we are now encountering the problem that approximately 80% of the samples are testing negative for COVID-19 and are thus being eliminated from the experiment.

The main comorbidities present in this study were hypertension (48.6%) and diabetes (28.6%). The data from the present study are similar to those of other recent experiments where the most prevalent comorbidities were also hypertension and diabetes [25-27]. These comorbidities are associated with the severity of COVID-19 and can significantly affect the prognosis of the disease [25,26]. Regarding transmission, 9 (25.5%) patients evaluated in this study had at least 1 person living in the same house who had already tested positive for SARS-CoV-2. The persons close to the other patients had not been tested or had already tested negative for SARS-CoV-2. According to Guan et al. [3], the transmission of SARS-CoV-2 occurs mainly between family members, including relatives and friends living in the same residence, even when they are asymptomatic.

The most frequent symptom presented by the patients on day 0 was coughing (74.3%), followed by loss of taste (54.3%), dyspnea (51.4%), hyposmia (48.6%), sore throat (22.9%), and body pain (22.9%). In other studies [25,26], the most prevalent symptom was fever. With the exception of fever, the prevalence of other symptoms was similar to what was found in the literature. In a previous systematic review [25], the prevalence of fever, coughing, fatigue, and dyspnea symptoms was 85.6%, 65.7%, 42.4%, and 21.4% respectively. In another study [26] the most prevalent clinical symptom was fever (91.3%) followed by coughing (67.7%), fatigue (51.0%), and dyspnea (30.4%). In the present study, all patients had some relief from symptoms during the 8 days, especially coughing, for both groups. In general, there was no difference in symptom relief between the 2 study groups. Loss of taste was the only oral manifestation evaluated by the present study, and was present in 54.3% of the sample on day 0. Recent studies have reported other oral manifestations related to COVID-19 [28], such as ulcerative lesions on the tongue, palate, lip, and cheek. COVID tongue is an inflammatory disease that usually appears on the top and sides of the tongue. Some evidence suggests that it is associated with high levels of the inflammatory cytokine interleukin-6, which is positively regulated in severe COVID-19 disease [29-31].

The oral viral load may be associated with the severity of COVID-19 [16]; thus, antiseptics that have the ability to damage or destroy the lipid layer of the virus, such as H_2_O_2_, have the potential to reduce the viral load of infected individuals, thereby decreasing the severity of symptoms [16,32]. Considering this, and the possible relationship between the viral load and the symptoms of fever, coughing, dyspnea, and sore throat, the relief of these symptoms over days 0-2 and 2-4 was evaluated. Since there was no relevant sample size for fever, it was excluded from this analysis. Dyspnea abated for 75.0% of the experimental group patients between days 0 and 2; this relief occurred for 30.0% of the placebo group in the same period. However, this possible effect must be demonstrated with a larger sample size, since it was not statistically significant. Recently, a study finding an association between H_2_O_2_ mouthwash use and the viral load of COVID-19 patients was published [33], but the results seem inconclusive due to the small sample size. That study, which did not find effectiveness for H_2_O_2_ solution used only as a mouthwash, is different from the present study that used a mouthwash and nasal spray.

The solutions did not reduce hospitalization time, which was 3.86± 1.60 days for the experimental group and 4.15± 1.77 days for the control group after starting solution use. Both groups continued using the solutions for the predetermined time even after discharge.

H_2_O_2_ has been used in dentistry for more than 70 years. In some situations, H_2_O_2_ at concentrations below 3% was used daily for up to 6 years, causing occasional and transient irritating effects only in a small number of individuals [21]. In the present study, which used concentrations of 0.5% (nasal spray) and 1.0% (gargle), few patients reported adverse effects after using the solutions. The most common effects were a burning sensation in the throat and in the nose, demonstrating its safety for use in low concentrations for 7 days. Some effects, such as nasal burning, diminished over time. Thus, the prolonged use of H_2_O_2_ for a longer period than in the present study still deserves further research.

In conclusion, H_2_O_2_ as a mouthwash (1.0%) and nasal spray (0.5%) is safe to use by patients. Some improvement trends in dyspnea could be observed. However, there is insufficient evidence to demonstrate that H_2_O_2_ is effective as an auxiliary treatment for hospitalized COVID-19 patients.

## Figures and Tables

**Table 1.** Demographic characteristics of patients at baseline, in absolute and relative values

Characteristics	Total (n=35)	Experimental (n=18)	Placebo (n=17)
Gender			
Men	22 (62.9)	9 (40.9)	13 (59.1)
Women	13 (37.1)	9 (69.2)	4 (30.8)
Age (yr)			
≤35	4 (11.4)	2 (50.0)	2 (50.0)
36-59	20 (57.1)	8 (40.0)	12 (60.0)
≥60	11 (31.4)	8 (72.7)	3 (27.3)
Race			
White	24 (68.6)	13 (54.2)	11 (45.8)
Non-White	11 (31.4)	5 (45.4)	6 (54.6)
Education level			
Completed high school	30 (85.7)	16 (53.3)	14 (46.7)
University education (complete/incomplete)	5 (14.3)	2 (40.0)	3 (60.0)
Family income (Brazilian reais)			
≤3,162.00	22 (62.9)	9 (40.9)	13 (59.1)
>3,162.00	13 (37.1)	9 (69.2)	4 (30.8)
No. of people in the same residence			
None	3 (8.6)	1 (33.3)	2 (66.7)
1	10 (28.6)	6 (60.0)	4 (40.0)
2	8 (22.9)	4 (50.0)	4 (50.0)
3	8 (22.9)	4 (50.0)	4 (50.0)
≥4	6 (17.1)	3 (50.0)	3 (50.0)
People who tested positive in the same residence			
None	24 (70.6)	11 (45.8)	13 (54.2)
1	9 (25.5)	7 (77.8)	2 (22.2)
≥2	1 (2.9)	0 (0.0)	1 (100)
Comorbidities			
Cardiac	3 (8.6)	0 (0.0)	3 (100)
Respiratory	3 (8.6)	3 (100)	0 (0.0)
Diabetes	10 (28.6)	6 (60.0)	4 (40.0)
Hypertension	17 (48.6)	9 (52.9)	8 (47.1)

Values are presented as number (%).

**Table 2.** Frequency of symptoms each day, in absolute and relative values

Symptoms	Day 0 (baseline)		Day 2		Day 4		Day 6		Day 8	
	H_2_O_2_	Control	H_2_O_2_	Control	H_2_O_2_	Control	H_2_O_2_	Control	H_2_O_2_	Control
Fever	-	-	-	1 (6.3)	1 (5.6)	-	1 (5.6)	-	-	-
Cough	13 (72.2)	13 (76.5)	13(72.2)	9 (56.2)	8 (44.4)	7 (41.2)	8 (44.4)	3 (18.7)	8 (44.4)	3(18.7)
Hyposmia	6 (33.3)	11 (64.7)	6 (33.3)	9 (56.2)	5 (27.8)	8 (47.1)	6 (33.3)	6 (37.5)	4 (22.2)	4 (25.0)
Loss of taste	7 (38.9)	12 (70.6)	7 (38.9)	10 (62.5)	7 (38.9)	6 (35.3)	6 (33.3)	5 (31.2)	5 (27.8)	4 (25.0)
Dyspnea	8 (44.4)	10 (58.8)	6 (33.3)	9 (56.2)	3 (16.7)	4 (23.5)	1 (5.6)	3 (18.7)	-	2 (12.5)
Sore throat	5 (27.8)	3 (17.7)	-	-	-	-	-	-	1 (5.6)	-
Body pain	3 (16.7)	5 (29.4)	2 (11.1)	3 (18.7)	-	3 (17.6)	-	2 (12.5)	-	1 (6.2)

Values are presented as number (%). H_2_O_2_, hydrogen peroxide.

**Table 3.** Absolute value and rate of symptomatic individuals^1^

Variables	Total with symptoms on day 0		Day 0-2		Day 2-4		Day 4-6	
	H_2_O_2_	Control	H_2_O_2_	Control	H_2_O_2_	Control	H_2_O_2_	Control
Coughing	13	13	4 (30.8)	7 (53.8)	6 (46.1)	5 (38.5)	1 (7.7)	2 (15.4)
Dyspnea	8	10	6 (75.0)	3 (30.0)	2 (25.0)	7 (70.0)	2 (25.0)	1 (10.0)
Sore throat	5	3	5 (100)	3(100)	0 (0.0)	0 (0.0)	0 (0.0)	0 (0.0)

Values are presented as number (%). H_2_O_2_, hydrogen peroxide.

1 On day 0 who experienced relief (but not necessarily complete elimination) of the symptoms of coughing, dyspnea, and sore throat over the first 6 days, starting on day 0.

**Table 4.** Frequency of adverse effects on each day: value and rate

Variables	Day 2		Day 4		Day 6	
	H_2_O_2_	Control	H_2_O_2_	Control	H_2_O_2_	Control
Burning mouth	1 (5.6)	-	-	1 (5.9)	-	-
Burning throat	4 (22.2)	1 (6.2)	-	2 (11.8)	1 (6.7)	-
Unpleasant taste of food after use	-	-	-	-	-	-
Feeling of thick tongue	-	-	3 (18.7)	-	1 (6.7)	-
Perceptible change in mucosa	-	-	-	-	-	-
Nasal burning	3 (16.7)	1 (6.2)	2 (12.5)	1 (6.2)	1 (6.7)	-

Values are presented as number (%). H_2_O_2_, hydrogen peroxide.
